# Long non-coding RNA HOTAIR as a competitive endogenous RNA to sponge miR-206 to promote colorectal cancer progression by activating CCL2

**DOI:** 10.7150/jca.42308

**Published:** 2020-05-18

**Authors:** Jia Shengnan, Xie Dafei, Jin Hua, Fan Sunfu, Wang Xiaowei, Xu Liang

**Affiliations:** Zhejiang Hospital, Hangzhou, 310013, China.

**Keywords:** Long noncoding RNA HOTAIR, CRC, mircroRNA-206, CCL2, Prognosis

## Abstract

Colorectal cancer (CRC) is one of the common malignant tumors, the incidence of which is on rise. LncHOTAIR, considered as an oncogene, contributed to the progression of a lot of cancers. However, the molecular mechanism and biological functions of the HOTAIR/miR-206/CCL2 axis have not been reported before. Here, our research aimed to explore HOTAIR/miR-206/CCL2 axis in CRC to demonstrate its role in predicting the poor prognosis of CRC. LncHOTAIR, miR-206 and CCL2 mRNA were detected in CRC tissues and cells by RT-PCR. The interactions among LncHOTAIR, miR-206 and CCL2 were explored by luciferase reporter assay, qRT-PCR, western blot and RNA interfere. Flow Cytometry Cell Analysis was performed to detect cell cycle and apoptosis as well as colony assay was prepared to test the cell proliferation. Immunohistochemical analysis was used to detect the CCL2 protein in CRC tissues. In our study, silence of LncHOTAIR by RNA interference could suppress the proliferation, migration and invasion of CRC cells. Mechanistically, LncHOTAIR downregulated miR-206 abundance which indicated that LncHOTAIR was considered as a competing endogenous RNA (ceRNA) by directly sponging miR-206 in CRC cells. In addition, further exploration suggested that miR-206 could inhibit the function of the downstream CCL2, the expression of which was repressed by LncHOTAIR/miR-206 signaling. Furthermore, we verified that the overexpression of CCL2 attenuated CRC cell proliferation, migration, invasion. Overall, this study firstly elucidated that LncHOTAIR played as oncogene in CRC via directly sponging miR-206 to activate the downstream CCL2, which would be considered as the novel therapeutic target in CRC.

## Introduction

Colorectal cancer (CRC) is one of the common malignant tumors, the incidence of which is on rise 1, 2. At present, about 1.4 million patients worldwide are diagnosed with CRC, nearly seven hundred thousand of who die of the disease 3. Although the means of advanced antitumor therapy are constantly updated, the regulatory mechanisms in CRC progression are still obscure.

Current studies have found that long chain non coding RNA (LncRNA) plays an important role in tumor progression. LncRNA transcribed by RNA polymerase II, which has obvious spatio-temporal expression specificity, lacks significant open reading frame, widely existing in nucleus and cytoplasm, so as not to participate in or rarely participate in protein coding 4. LncRNAs, which spongdes miRNA, play the role of oncogenes or tumor suppressor genes in the development of tumor through regulating the proliferation, invasion, apoptosis and drug resistance of cancer cells 5. Emerging evidence has exerted a lot of LncRNAs take part in the CRC progression, including homeobox transcript antisense inter-genic RNA (HOTAIR)^6^. Previous research showed HOTAIR overexpressed in the proximal and distal colon cancer and associated with lymph nodes metastasis 7. It could sponge miR-203a-3p 8, miR-218 9, miR-326 10, miR-197 11 and miR-206 to exhibit its function of oncogene in regulation of Wnt, PI3K and NF-κB signaling in CRC. MicroRNA (miRNA), like miR-203, miR-206 and miR-326, can target mRNA, by base complementary pairing to degrade mRNA or inhibit its protein translation, which plays an important role in regulating gene expression, maintaining cell morphology and controlling cell cycle. The expression level of miRNA in tumor tissues is often different from that in normal tissues, which may not only participate in tumorigenesis as a carcinogenic factor, but also play an anti-tumor role as a tumor suppressor. Therefore, it is of great significance to clarify the role of miRNA especially the interaction between LncRNA and miRNA in the diagnosis and treatment of tumors including CRC.

Some researches focused on the HOTAIR/miR-206 axis in head and neck squamous cell carcinoma, ovarian cancer and breast cancer, however few researches concentrated on its function in CRC. miR-206 has been considered as the tumor suppressor in variety cancers, especially in CRC. In our study, we found that expression of HOTAIR was significantly increased in CRC tissues and cells. Loss function of HOTAIR repressed the proliferation and invasion and provoked the apoptosis of CRC cells, the deep mechanism of which may be it sponged miR-206, to activate the downstream CCL2, finally leading to the promotion of CRC progression. Thus, we intended to uncover the role of HOTAIR/miR-206/CCL2 axis in cell migration and invasion of CRC and the possible underlying mechanisms. This result explored the new mechanism of HOTAIR in CRC, which would indicate the new therapeutic target in clinical application.

## 2. Materials and methods

### 2.1 Tissue samples collection

All the 32 CRC tissues and paired normal tissues, from the patients who has not gained therapy like preoperative chemotherapy or radiotherapy before, were obtained from specimen repository of Zhejiang Hospital from 2012-2017. Inclusion criteria included any case which were diagnosed and treated for stage II-IV colorectal cancer. Exclusion criteria included cases who received chemotherapy or radiotherapy as well as cases with missing data. Specimen has been frozen with liquid nitrogen after surgery or biopsy and kept at -80℃ until later use. This study achieved the permission from the ethics committee of Zhejiang Hospital. Prior to the usage of these clinical materials, every patient offered the informed consent. The different stage of CRC (Stage II-IV) was referred to previous article 12-14.

### 2.2 Cell culture and transfection

The human CRC cells lines including SW620, SW480, HCT116, HT29, LoVo, and the normal colon cell line, NCM460 were purchased from the Chinese Academy of Sciences (Shanghai Cell Bank). Cells were cultured in a complete RPMI1640 medium (RPMI1640; Gibco, Grand Island, NY, USA) containing 10% fetal bovine serum (FBS; Gibco, Grand Island, NY, USA) and 1% double antibody (ElifeBio, Hangzhou, China), in a constant temperature incubator at 37℃ with humidified 5% CO2 atmosphere. When the cell density was more than 80%, trypsin was used for digestion and subculture and seed plate. Cell transfection referred to protocol of Lipofectamine 2000 (Invitrogen, Grand Island, NY). The small interference RNAs plasmid (siRNA) of HOTAIR and CCL2 according to the manufacturer's protocol as well as small interference RNA plasmid of CCL2 were purchased from Genechem (Shanghai, China). The miR-206 inhibitor, miR-206 mimics, mimic NC and negative control (NC) were synthesized by GenePharma (Shanghai, China). Transfection efficiency was determined by qRT-PCR.The detailed sequencings were listed in Table S1.

### 2.3 Quantitative real-time polymerase chain reaction (qRT-PCR)

After 48 hours of transfection and culture, the total RNA was extracted by Trizol reagent (Invitrogen, Carlsbad,USA) for qRT-PCR detection. The SYBR Green PCR Master One-Mix kit (Transgen, Beijing, China) and cDNA Reverse Transcriptase Kit (TAKARA, Beijing, China) were purchased from ElifeBio (Hangzhou, China). Real-time PCR 7300 System (Applied Biosystems, Foster City, USA) was used for qRT-PCR detection. The reaction conditions were as follows: pre-denaturation at 95℃ for 5 min, denaturation at 95℃ for 10 s, annealing at 60℃ for 30 s, and 35 cycles. Each group was repeated 3 times. Relative quantification of RNA expression was calculated using the 2-ΔΔCT method. All the primers of HOTAIR, miR-206, CCL2 and GAPDH were shown in Table S.

### 2.4 Cell proliferation analysis

The transfected cells were inoculated on 96-well plate according to the density of 10^4^ / well, and the cells were cultured for 24 h, 48 h, 72 h and 96 h, as well as the cell viability was detected. After the cell culture medium was removed, the pre-configured cell counting kit-8 (CCK-8, ElifeBio, Hangzhou, China) reagent was added, before the cell culture plate was placed in a constant temperature incubator for 30 min. The cell culture plate was placed in an enzyme labeling instrument to read the absorbance value at 490 nm measured using a microplate reader (Thermo Scientific). At last the proliferation ability of the cell was calculated.

### 2.5 Cell colony assays

Cell colony assay was prepared referring to a previous article 11.

### 2.6 Flow cytometry cell cycle and apoptosis analysis

The transfected cells were inoculated on the 6-well plate at the density of 10^6^ / well, and the apoptosis of the cells was detected after 24 hours of culture. The cells were collected by trypsin digestion and centrifugation, mixed with the binding solution, and then added with the Annexin V-FITC reaction reagent at the light avoidance reaction 10 min the propidium iodide (PI) staining solution again avoiding the light reaction 10 min, and finally adding the binding solution, the percentage of apoptosis was detected. The cell cycle distribution was analyzed by flowcytometry (FACS Calibur, BD Biosciences).

### 2.7 Cell invasion assays

Cell invasion assays were measured by transwell chamber with Matrigel (8-μm pore size; BD Biosciences, USA) according to the manufacturer's protocol. The detail of the assay referred to the previous study 15. The invaded cells were stained with 1% crystal violet, photographed using a microscope with a 10× objective (total magnification 100×) and counted. Experiments were performed in triplicate.

### 2.8 Western-blot analysis

The expression of cell protein was detected after 24 hours of culture. RIPA lysate was used to extract the total cell protein and determine the total protein. Each group was treated with 100 μg protein for electrophoresis, as well as PVDF membrane was used for membrane transfer sealed at room temperature for 1 hour, then the membrane was incubated overnight with the first antibody at low temperature, and horseradish peroxidation was added the next day. The second antibody was incubated for 1 hour, and then the chromogenic solution was added for color development and photographed and analyzed. The antibody was listed in the Table S2.

### 2.9 Immunohistochemical analysis

After cut into 4-µm sections Tissues were deparaffinized and treated with EDTA (pH 9.0) to antigen retrieval in a microwave for 20 min. Autostainer Link 48 machine (Dako, Denmark A/S, Denmark) was performed for staining. The detail of the assay referred to the previous study 16. The antibody was listed in the Table S2.

### 2.10 Statistical analysis

All experimental data from experiments were analyzed by GraphPad Prism 8.0 and results were shown as mean ± SD (standard deviation, SD). Comparisons between two groups were made by Student's t-test. Multiple comparisons were made by One-way ANOVA. Survival analysis was analyzed via the Kaplan-Meier method. P < 0.05 was considered significant.

## Results

### 3.1 HOTAIR overexpression in CRC predicts the poor prognosis

**Figure 1:**
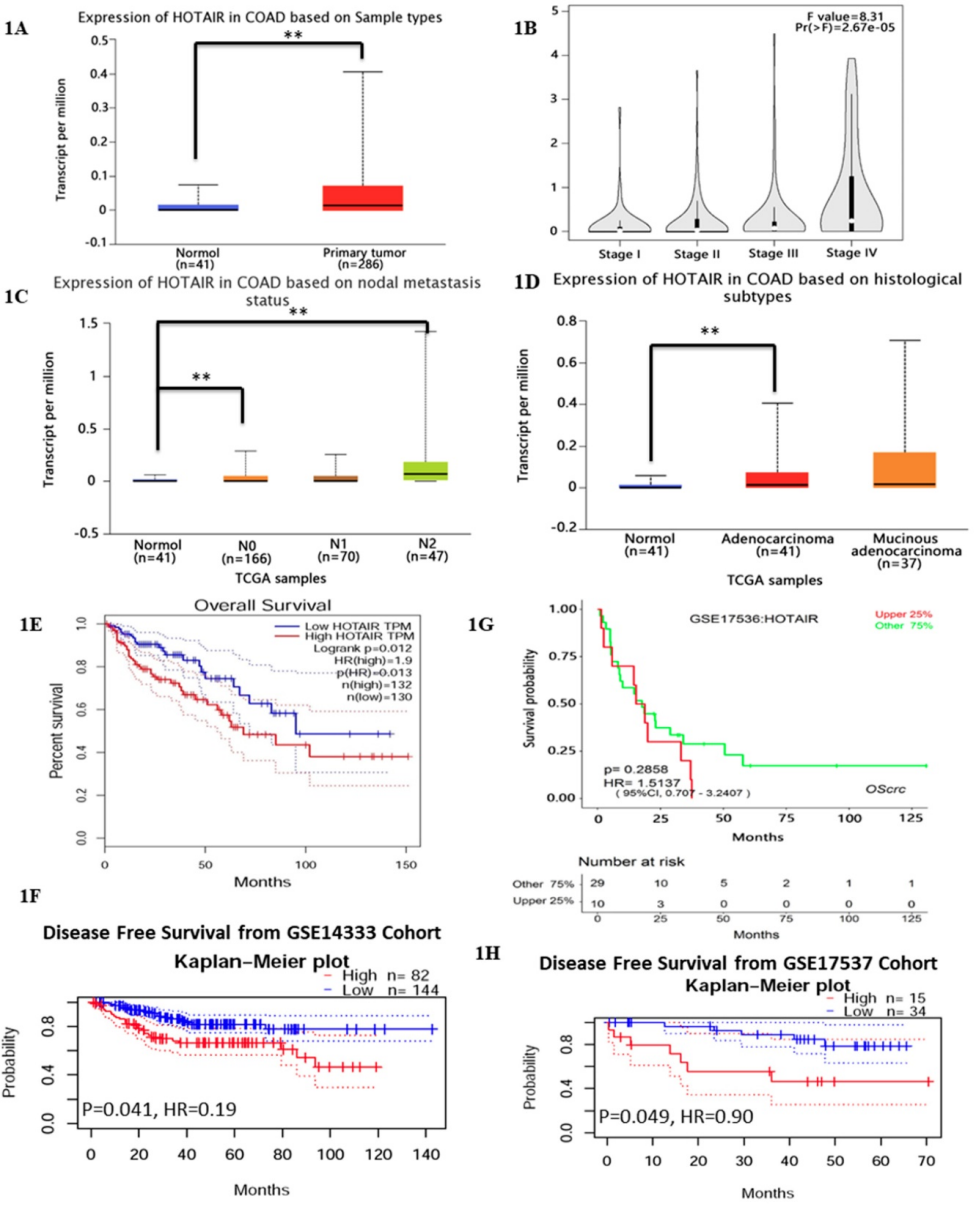
Exploring HOTAIR from the Cancer Genome Atlas (TCGA) in cancers by online tools “GEPIA”, “UALCAN” and “Survexpress”. **A.** The expression of lncRNA HOTAIR was analyzed by “UALCAN” and the data were from TCGA. The expression of lncRNA HOTAIR was upregulated in colon cancer tissue (n=286) and the paired normal colon tissue (n=41). **B.** The expression of lncRNA HOTAIR was further analyzed by “GEPIA”in different clinical stages and lncRNA HOTAIR was upregulated higher in stage IV and stage III than that in stage I and stage II. **C.** The expression of lncRNA HOTAIR in different nodal stages by“UALCAN”, and lncRNA HOTAIR was upregulated higher in N IV and stage III than that in stage I and stage II. **D.** The expression of lncRNA was upregulated in adenocarcinoma than normal tissue analyzed by “UALCAN”. **E.** and **F.** The high expression of LncRNA HOTAIR indicated the lower overall survival analyzed by “GEPIA”(E) and “Survexoress”(F) *, P<0.05. **, P<0.01. **G.** and **H.** The high expression of LncRNA HOTAIR suggested the lower disease free survival analyzed by “PrognoScan” *, P<0.05. **, P<0.01.

**Figure 2:**
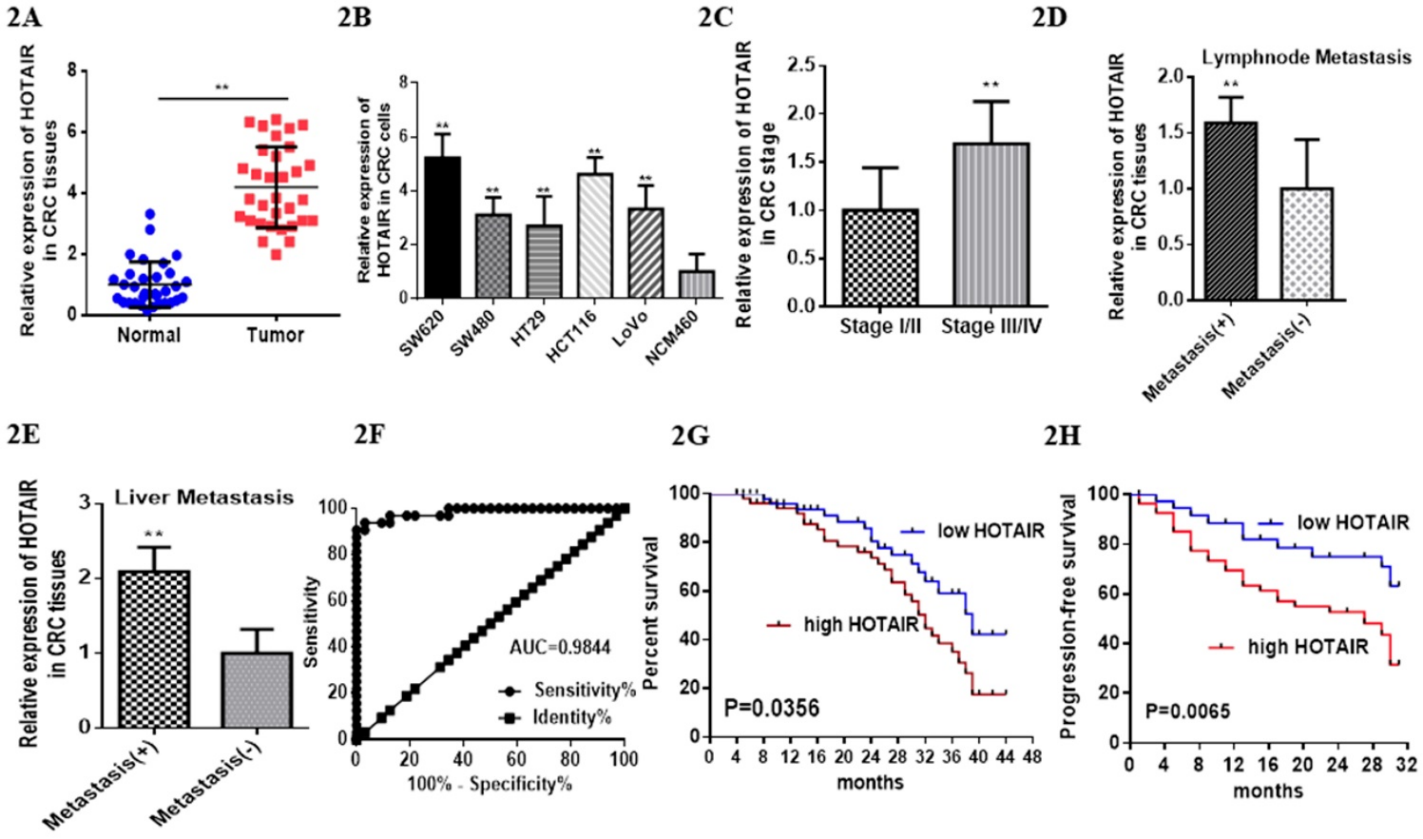
LncHOTAIR overexpression in CRC tissues. **A.** LncHOTAIR overexpressed in CRC tissues compared to normal tissues by qRT-PCR. **B.** LncHOTAIR overexpressed in CRC cells compared to normal cell by qRT-PCR. **C.** LncHOTAIR overexpressed in advanced stages (Stage I/II: 18; Stage III/IV: 14) of CRC tissues by qRT-PCR. **D.** LncHOTAIR overexpressed in CRC with lymphnode metastasis tissues (positive: 13; negative: 13) by qRT-PCR. **E.** LncHOTAIR overexpressed in CRC with liver metastasis tissues (positive: 14; negative: 18) by qRT-PCR. **F.** ROC curve analysis of LncHOTAIR expression in colorectal tissues. **G and H.** Kaplan-Meier survival curve indicated that LncHOTAIR overexpression in patients with CRC showed lower survival in overall survival** (G)** and progress in-free survival** (H)**. * P<0.05, ** P<0.01, compared with NC group.

Previous studies have reported HOTAIR overexpressed in various cancer types 17, which leads us to explore its function or its role in CRC. We searched HOTAIR from the Cancer Genome Atlas (TCGA) in cancers by online tools “GEPIA”, “UALCAN”and “Survexpress”, finding that expression of lncRNA HOTAIR was upregulated in colon cancer tissue (n=286) , especially the adenocarcinoma tissue (Fig. **1D**, p < 0.01) and the paired normal colon tissue(n=41) by “UALCAN” database (http://ualcan.path.uab.edu) (Fig. **1A**, p < 0.01) as well as upregulated higher in stage IV and stage III than that in stage I and stage II by “Gepia” database (http://gepia.cancer-pku.cn/) (Fig. **1B**, p < 0.01). High expression of HOTAIR also showed the severe nodal stage (Fig. **1C**, p < 0.01) as well as the overall survival which meaned the poor prognosis (Fig. **1E**, **1F**, p < 0.01). The cohort GSE 14333 (Fig. **1G**, p < 0.05) and GSE 17537 (Fig. **1H**, p < 0.05) from GEO database showed high expression of HOTAIR may suggest the poor disease free survival, which may be good proof of the validity of our result. In our research, from 32 CRC tissues and paired normal tissues, found HOTAIR also overexpressed in tumors in comparison with the normal (Fig. **2A**, p < 0.01). We also detected the HOTAIR expression in CRC cells to prove its high level of expression both in CRC tissues and cells (Fig. **2B**, p < 0.01). Moreover, we also found HOTAIR overexpressed in the CRC patients with stage III/IV compared with the stage I/II (Fig. **2C**, p < 0.01). Further exploration showed HOTAIR overexpression correlated with lymphnode metastasis (Fig. **2D**, p < 0.01) and liver metastasis (Fig. **2E**, p < 0.01). Furthermore, we aimed to explore the cut-off value of HOTAIR to establish its high or low expression in CRC. Our result found 1.967 was the threshold for HOTAIR (the relative mRNA levels of HOTAIR compared with GAPDH). Correspondingly mRNA levels of HOTAIR >1.967 was considered as “high” and those≤1.967 as “low” (Fig. **2F**, AUC=0.9844). We also found that the expression of HOTAIR mRNA significantly correlated with distant metastasis (P = 0.01), lymph node metastasis (P = 0.0001), MMR index (P = 0.034) and depth of invasion (P = 0.011) (**Table 1**).

Patients with high HOTAIR expression revealed poor prognosis, especially the lower overall survival time compared with the patients with low expression (Fig. **2G**, mean survival: 32.0 months vs 38.0 months). High expression of HOTAIR also predicted the poor progressin-free survival (Fig. **2H**). Furthermore, HOTAIR has been identified as the independent factor to impact the overall survival (**Table 2**).

### 3.2 Loss function of HOTAIR suppressed proliferation and invasion and provoked apoptosis in CRC cells

**Figure 3:**
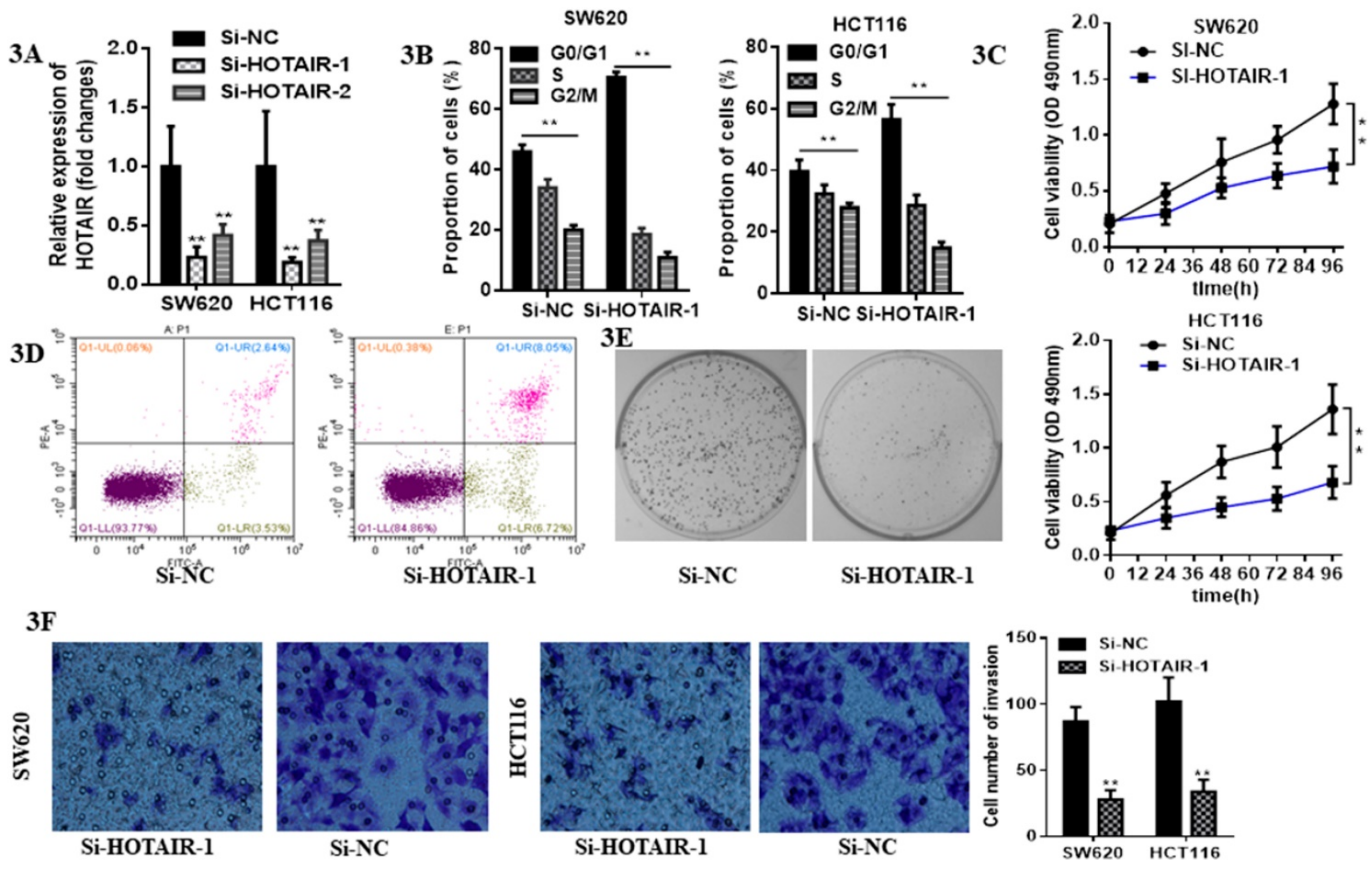
LncHOTAIR down-regulation inhibited proliferation and invasion of CRC cells. **A.** LncHOTAIR-1 and LncHOTAIR-2 were transfected in SW620 and HCT116 cells for silence and qRT-PCR was performed for validation. **B.** Flow cytometry assay was performed to explore the proportion of G0/G1 phase-arrested CRC cells as well as S and G2/M phase in LncHOTAIR-silenced group or control group in SW620 and HCT116 cells. **C.** CCK-8 assay was prepared to show that LncHOTAIR knockdown inhibited the ability of proliferation in SW620 and HCT116 cells. **D.** Flow cytometry assay was performed to show LncHOTAIR knockdown accelerated the apoptosis of SW620 cells. **E.** Inhibition of LncHOTAIR suppressed the colony formation of SW620 cells. **F.** LncHOTAIR silencing decreased invasive cell numbers via Transwell assay in SW620 and HCT116 cells. * P<0.05, ** P<0.01, compared with NC group.

Due to the higher expression of HOTAIR in HCT116 and SW620 than other cell lines, we selected these two cell lines for the further research. The expression of HOTAIR was obviously decreased by qRT-PCR analysis after transfection with either Si-HOTAIR-1 or Si-HOTAIR-2, but the former showed much more power (Fig. **3A**). Moreover, CRC cells, silenced HOTAIR-1, were blocked at G0/G1 phase by flow cytometry cell cycle analyses (Fig. **3B**). The CCK8 assay results uncovered that knockdown of HOTAIR could repress cell viability (Fig. **3C**). Loss function of HOTAIR elevate the apoptotic rate, which was observed in si-HOTAIR-1-transfected CRC cells by flow cytometry apoptosis assay (Fig. **3D**). Moreover, colony formation assay exhibited inhibition of LncHOTAIR suppressed SW620 cells proliferation (Fig. **3E**). Afterwards, Transwell assay further indicated the silenced of HOTAIR decreased invasive cell numbers, which revealed HOTAIR would contribute to CRC metastasis (Fig. **3F**).

### 3.3 HOTAIR sponged miR-206 to regulate the progression of CRC cells

**Figure 4:**
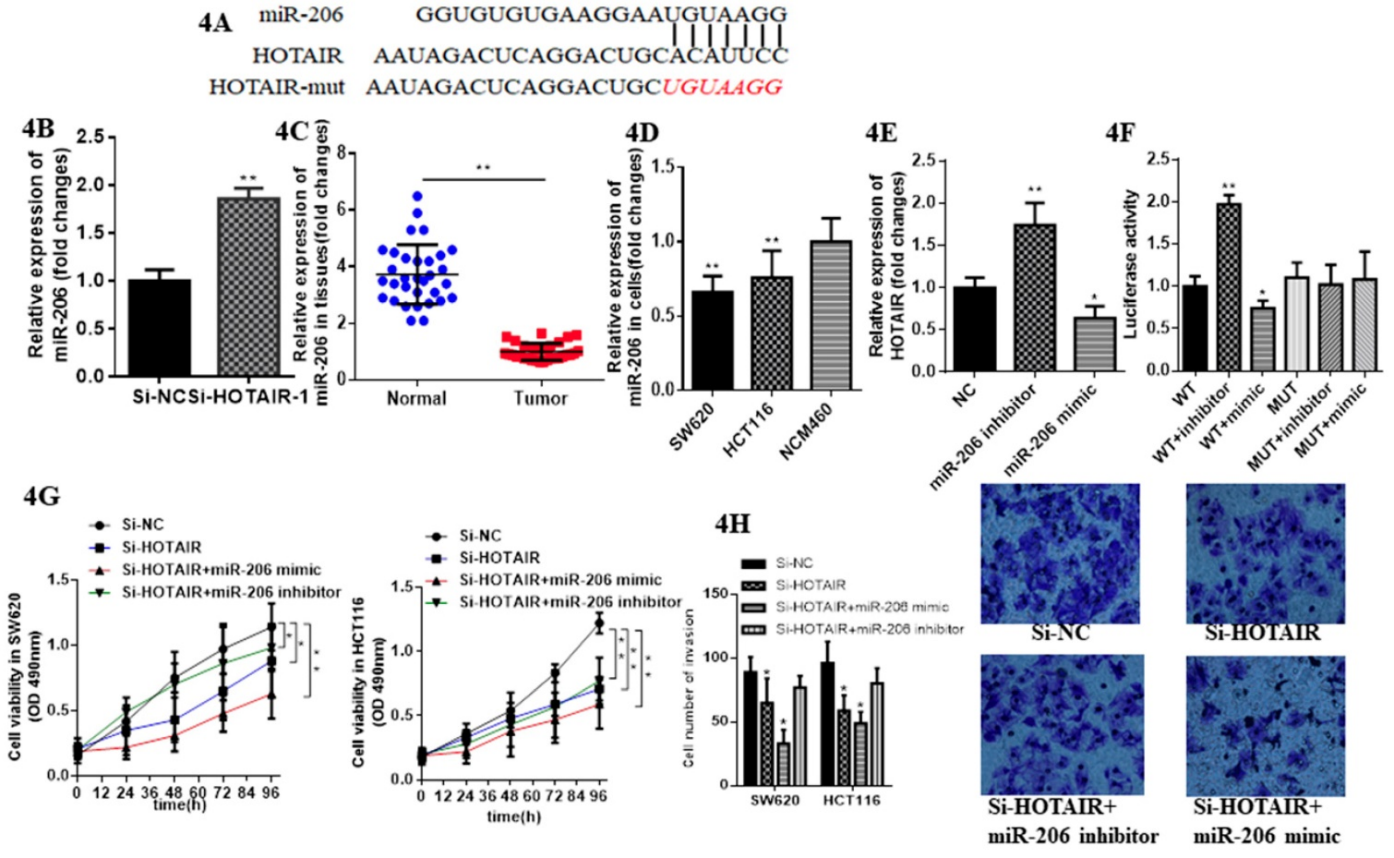
miR-206 suppresses function of LncHOTAIR in CRC cells. **A.** Bioinformatics analysis of HOTAIR and miR-206. **B.** The mRNA expression levels of miR-206 in SW620 cells were detected by qRT-PCR in LncHOTAIR-silenced group or control group. **C.** miR-206 showed low expression in CRC tissues compared to normal tissues by qRT-PCR. **D.** miR-206 showed low expression in CRC cells compared to normal cells by qRT-PCR. **E.** The mRNA expression levels of LncHOTAIR in SW620 cells were detected by qRT-PCR in miR-206 mimic group, inhibitor group or control group. **F.** Cell lysates in SW620 and HCT116 cells transfected with wild-type or mutant HOTAIR plasmid and cotransfected with miR-206 mimic or miR-206 inhibitor were assayed for luciferase activity. **G.** Cell proliferation in SW620 transfected with HOTAIR-silenced and cotransfected with miR-206 mimic, miR-206 inhibitor were tested by CCK-8 assay. **H.** Cell invasion ability in SW620 transfected with HOTAIR-silenced and cotransfected with miR-206 mimic or miR-206 inhibitor was tested by transwell assays. * P<0.05, ** P<0.01, compared with NC group.

Emerging evidence demonstrates lncRNAs considered as ceRNA could regulate miRNAs in various cancer. Therefore, we aimed to verify whether HOTAIR could exhibit this function in CRC. We identified miR-206 as a most potential candidate via bioinformatics analysis and bioinformatics database (miRDB, http://mirdb.org; segal lab, https://genie.weizmann.ac.il).Thus, we further aimed to detect their direct interaction (Fig. **4A**). We constructed luciferase reporter plasmids of HOTAIR. Moreover, HOTAIR-silenced resulted in the increased expression of miR-206 by qRT-PCR (Fig. **4B**). Meanwhile miR-206 may exert tumor suppressor in CRC due to its low expression in CRC tissues and cells (Fig. **4C**, **4D**). The expression of HOTAIR significantly decreased when SW620 transfected with the miR-206 mimic, whereas transfected with the miR-206 inhibitor repressed it significantly (Fig. **4E**). Luciferase reporter, transfected into SW620 cell, containing exact or mutant miR-206 binding sites was prepared for further detection of the bonding effect between HOTAIR and miR-206, which showed miR-206 mimic significantly decreased the luciferase activity of the wild-type HOTAIR reporter plasmid, as well as the miR-206 inhibitor significantly increased it. However this effect was counteracted when mutant HOTAIR reporter plasmid was transfected (Fig. **4F**). The CCK-8 assay showed cell proliferation in SW620 and HCT116 transfected with HOTAIR-silenced and cotransfected with miR-206 mimic was suppressed significantly compared with cotransfected with miR-206 inhibitor (Fig. **4G**). Furthermore, the invasion in SW620 and HCT-116 transfected with HOTAIR-silenced and cotransfected with miR-206 mimic was significantly suppressed, but increased cotransfected with the miR-206 inhibitor (Fig. **4H**). Taken together, these results conveyed that HOTAIR, functioned as the oncogene, could sponge miR-206 to provoke proliferation and invasion and suppress apoptosis in CRC cells.

### 3.4 CCL2 was a novel target of miR-206 in colorectal cancer cells

**Figure 5:**
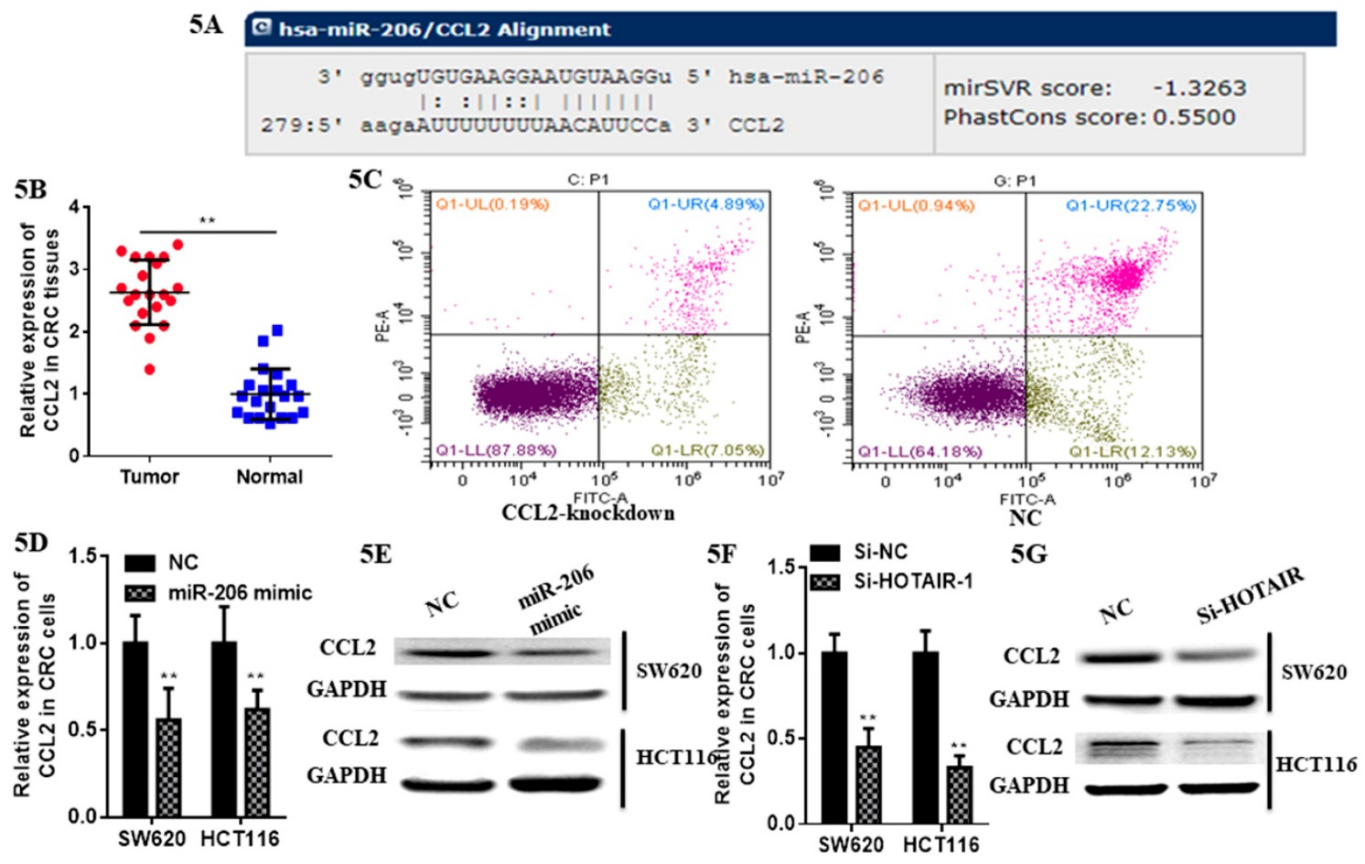
LncHOTAIR/miR-206 targeted CCL2 in CRC cells. **A.** Bioinformatics analysis of CCL2 and miR-206. **B.** CCL2 showed high expression in CRC tissues compared to normal tissues by qRT-PCR. **C.** Flow cytometry assay was performed to show CCL2 knockdown accelerated the apoptosis of SW620 cells. **D.** The mRNA level of CCL2 was detected in CRC cells transfected with miR-206 mimic by qRT-PCR assay. **E.** The protein level of CCL2 was measured in CRC cells transfected with miR-206 mimic by western blotting.** F.** The mRNA level of CCL2 was detected in CRC cells transfected with HOTAIR knockdown by qRT-PCR assay. **G.** The protein level of CCL2 was measured in CRC cells transfected with HOTAIR knockdown by western blotting. * P<0.05, ** P<0.01, compared with NC group.

**Figure 6:**
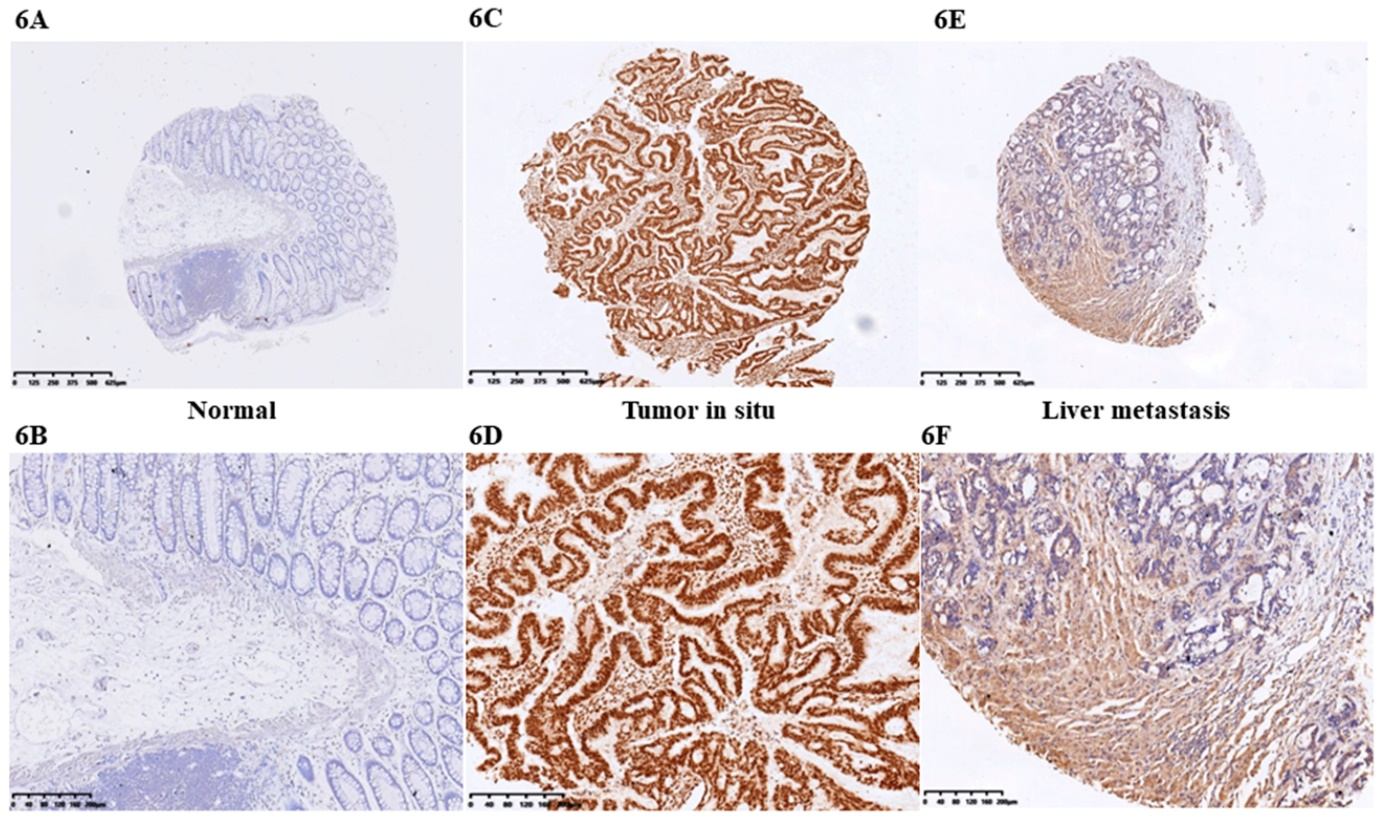
IHC staining of CCL2 in CRC tissues. **A.** CCL2 showed almost no staining in normal tissues (4X). **B.** CCL2 showed almost no staining in normal tissues (10X). **C.** CCL2 showed strong staining in tumor tissues (4X). **D.** CCL2 showed strong staining in tumor tissues (4X). **E.** CCL2 showed moderate staining in CRC liver metastasis tissues (4X). **F.** CCL2 showed moderate staining in CRC tissues (10X) by IHC.

At first we selected the gene correlated with proliferation, invasion and apoptosis targeted by miR-206, which was predicted by (http://www.microrna.org/microrna). According to bioinformatics data, we screened out CCL2. Analysis of the 3'UTR sequence of the CCL2 gene by TargetScan revealed a putative miR-206 binding site (Fig. **5A**). Then we explored the role of CCL2 in CRC, that it overexpressed in CRC tissues compared with normal ones by qRT-PCR (Fig. **5B**). Moreover, the IHC staining in normal tissues, distinguished from the positive cells and the strong staining, was stronger in normal tissues compared with the almost no staining in CRC situ tissues especially in liver metastasis tissues (Fig. **6**). Due to the low expression of CCL2 in CRC tissues, we sought to explore the its role in regulation of phenotype of CRC. Knockdown of CCL2 mRNA could significantly accelerated the apoptosis of SW620 cells (Fig. **5C**). miR-206 mimic could restrain the CCL2 mRNA and protein (Fig. **5D**, **5E**), in addition, knockdown of HOTAIR significantly augmented mRNA and protein level of CCL2 in colorectal cancer cells (Fig. **5F**, **5G**). Thus, we concluded that CCL2 was a direct target of miR-206 and positively modulated by HOTAIR in CRC cells.

### 3.5 miR-206/CCL2 mediated suppressive roles of HOTAIR on cell viability, apoptosis, invasion on colorectal cancer cells

**Figure 7:**
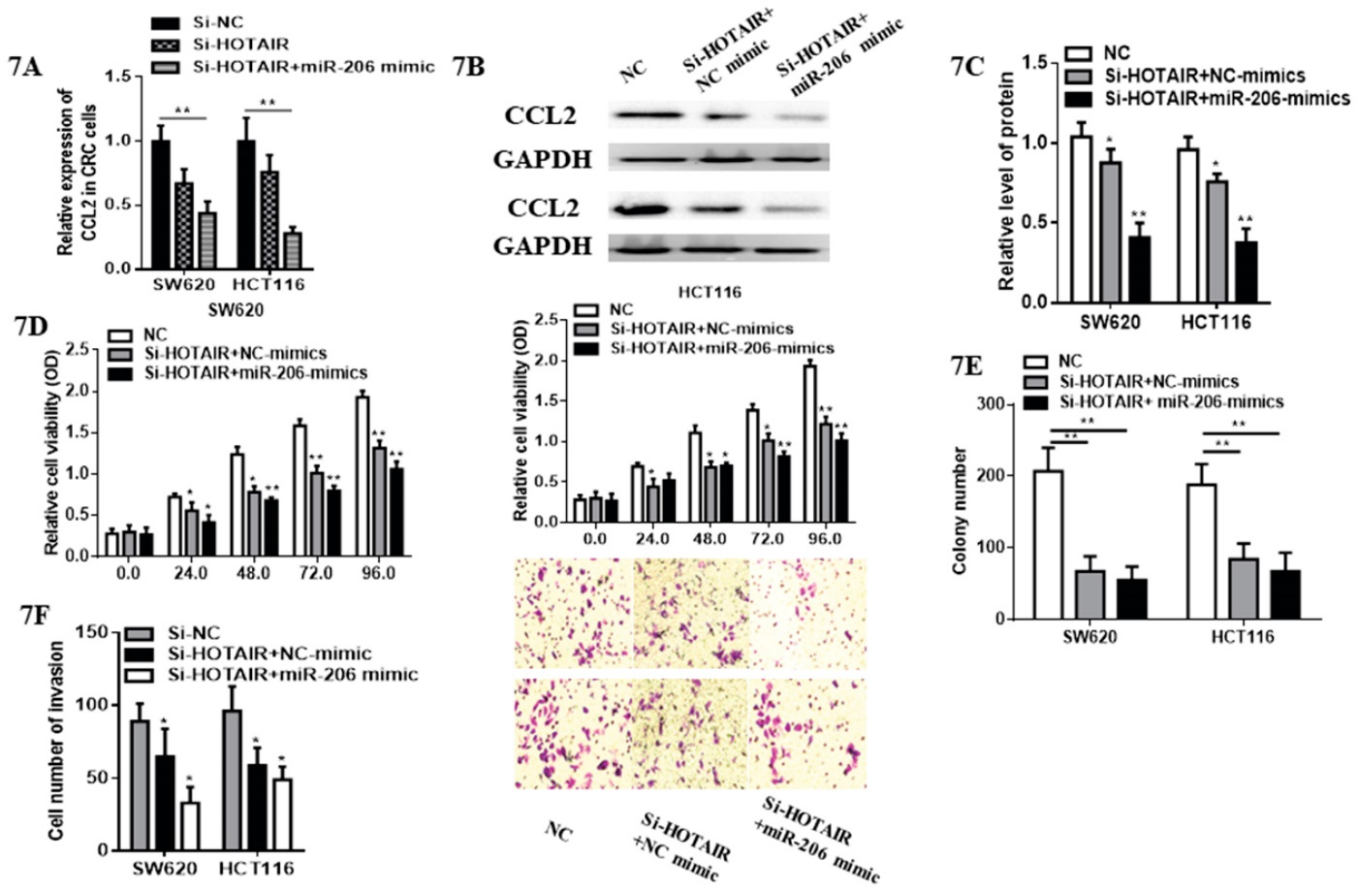
miR-206 attenuated pro-tumorous effects of HOTAIR in colorectal cancer. **A.** miR-206 overexpression decreased the mRNA expression of CCL2 in HOTAIR-knockdown colorectal cancer cells. **B.** miR-206 overexpression reduced the protein expression of CCL2 in HOTAIR-knockdown colorectal cancer cells. **C.** Quantify the relative expression intensity of CCL2 protein. **D.** CCK-8 assays suggested that miR-206 could effectively accelerate the inhibitory effects of HOTAIR knockdown on proliferation of CRC cells. **E.** miR-206 accelerated the inhibitory ability of the colony formation in HOTAIR-knockdown CRC cells. **F.** miR-206 enhanced the suppressive effects of HOTAIR-knockdown on invasion of CRCcells. * P<0.05, ** P<0.01, compared with NC or NC-mimic group.

Although cell proliferation in SW620 transfected with HOTAIR-knockdown was significantly suppressed compared with control group. Subsequently, we validated whether miR-206 could exert its suppressive effects on CRC progression through HOTAIR/miR-206/CCL2 axis. Notably, miR-206 overexpression decreased the expression of CCL2 mRNA and protein (Fig. **7A, 7B, 7C**) in HOTAIR-knockdown CRC cells. Moreover, miR-206 overexpression could effectively accelerate the inhibitory effects of HOTAIR knockdown on proliferation of CRC cells (Fig. **7D**). Functionally, miR-206 attenuated the colony formation in HOTAIR-knockdown CRC cells (Fig.**7E**). Furthermore, cell invasion of SW620 and HCT116 was significantly suppressed by HOTAIR-knockdown, additionally, miR-206 overexpression could accelerate this progression (Fig. **7F**). Therefore, our results showed that HOTAIR could exert its oncogenesis effects on the growth and invasion of CRC via HOTAIR/miR-206/CCL2 axis.

## Discussion

The purpose of our research was to identify the role of HOTAIR/miR-206/CCL2 axis in regulation of CRC as well as the therapeutic target in clinical practice. Previous studies have confirmed that HOTAIR, as an oncogene, has taken part in the proliferation, metastasis and epithelial mesenchymal transformation in CRC 18, 19. Moreover, HOTAIR was overexpressed in various cancers such as breast cancer 20, pancreatic cancer 21, esophageal squamous cell carcinoma 22, hepatocellular carcinoma (HCC) 23 and CRC 24. Furthermore, from a clinical trial, circulating HOTAIR DNA of patients with CRC was significantly higher than that of healthy people, the sensitivity and specificity of which for the diagnosis of tumors were 67% and 92.5% respectively 25. Based on the pathological specimen, our data supported previous study that the higher expression level of HOTAIR in CRC than healthy people. Due to the complicated function of which can not only facilite CRC cells proliferation through corresponding molecular signaling pathways 10, but also sponge the microRNA to accelerate the tumor progression [26]or to make the tumor cells resistant to 5-FU 9. So HOTAIR exhibiting the oncogenesis function would be mainly due to sponging the target miRNA.

Based on the bioinformatics database (miRDB, http://mirdb.org) as well as referred to the high biological score, we speculated that miR-206 may be sponged by HOTAIR.miR-206, first found in skeletal muscle as well as considered to be related to the physiological and pathological process of skeletal muscle, has been identified as a tumor suppressor in many human malignancies, including breast cancer 27, renal cancer 28, lung cancer 29, colorectal cancer^30^, and gastric cancers 31. Up-regulation of miR-206 expression can inhibit tumor progression by blocking cell cycle, promoting apoptosis, inhibiting cell metastasis and angiogenesis 32. Meanwhile, miR-206 can reduce the resistance of CRC cells to 5-FU by targeting the expression of Bcl-2 30. In CRC, miR-206 expression was significantly lower in CRC tissues than adjacent normal tissues which were also clarified in our study. Mechanically, it directly targeting FMNL2 markedly suppressed cell proliferation and invasion in CRC 32. It's interesting that we verified HOTAIR could act as a competitive endogenous to sponge miR-206 to enhance ability of proliferation and invasion in CRC cells. There are three studies in head and neck squamous cell carcinoma 33, ovarian cancer 34, breast cancer 35 but CRC focusing on the repressing role of HOTAIR to sponge miR-206. The early one in the three was to identify HOTAIR sponging miR-206 could activate bcl-w to facilitate the cell proliferation 35. The following researches confirmed that HOTAIR negatively regulated miR-206 to activate STS2 33 to promote cancer invasion of head and neck squamous cell carcinoma as well as to activate CCND1 and CCND2 34 to stimulate the proliferation, and invasion of ovarian cancer cells. It is meaningful to explore the HOTAIR/miR-206 axis in CRC.

Our study has confirmed the role of HOTAIR as oncogene in CRC tissues which associated with liver metastasis and advanced stage predicted the poor prognosis of CRC. Loss function of HOTAIR by RNA interference could suppress the proliferation, migration and invasion of CRC cells. Mechanistically, HOTAIR suppressed miR-206 abundance which indicated that HOTAIR directly sponged miR-206 in CRC cells. Further detection suggested that miR-206 could repress the function of the downstream CCL2, which also considered as the oncogene in CRC 36. From IHC results, CCL2 indeed showed high expression in tumor tissues, especially liver metastatic tissues. Previous study also has demonstrated miR-206 could suppress CCL2/VEGFA to inhibit the progression of normal fibroblasts into cancer-associated fibroblasts 36. CCL2 plays a crucial role in the recruitment of inflammatory macrophages to the tumor site and become TAMs that are suggested to enhance tumor malignancy in CRC 37. Furthermore, CCL2 overexpression induces tumor angiogenesis via TAMs in gastric cancer 38. Interestingly CCL2 accelerated the CRC liver metastasis via Wnt/β-catenin signaling in animal model 39. All these proved that CCL2 plays a role as an oncogene in the process of carcinogenesis and development. However, as no research has verified the function in the biological role the progression and metastasis in CRC, miR-206/CCL2 axis is poorly understood in CRC. We indeed found CCL2 showed low expression in normal colorectal tissues but tumor tissues as well as loss function of HOTAIR or overexpression of miR-206 would repress the activation of CCL2. In contrast to the previous research, we not only have identified HOTAIR sponged the miR-206 in CRC, but verified miR-206 suppressed the CCL2. This axis may exhibit the important role in CRC.

In conclusion, our research has illustrated that HOTAIR overexpressed in CRC which predicted poor outcomes of patients. HOTAIR provoked proliferation and invasion of CRC through sponging miR-206 and repression of CCL2. Hence, our study indicated that HOTAIR/miR-206/CCL2 would be the novel therapeutic target in CRC.

## Supplementary Material

Supplementary tables.

## Figures and Tables

**Table 1:** Relationship between HOTAIR and clinicopathological factors of colorectal cancer patients

Characteristic	Number	Expression of HOTAIR		P value
		High	Low	
**Ages (years)**				
<60	17	9	8	0.98
≥60	15	8	7	
**Sex**				
male	16	8	8	0.72
female	16	9	7	
**Tumour diameter (cm)**				
≤5	15	7	8	0.49
>5	17	10	7	
**Histological differentiation**				
Well	14	6	8	0.30
Poor	18	11	7	
**Lymph node metastasis**				
Positive	14	11	3	0.011*
Negative	18	6	12	
**Distant metastasis**				
Positive	13	12	1	0.0002**
Negative	19	5	14	
**MMR index**				
dMMR	9	8	1	0.0112*
pMMR	23	9	14	
**Depth of invasion**				
T1+T2	18	6	12	0.011*
T3+T4	14	11	3	

**Table 2:** Univariate and multivariate analyses of clinicopathologic factors for OS in colorectal cancer patients

Variable	Univariate analysis				Multivariate analysis		
	HR	95% CI	*P* value		HR	95% CI	P value
Age (≥60 vs. <60 years)	0.683	0.238∼3.175	0.758				
Sex (Male vs. Female)	0.786	0.285∼2.233	0.657				
Tumour diameter (≥5 vs. <5 cm)	0.866	0.365∼2.015	0.743				
Depth of invasion (T1+T2 vs. T3+T4)	1.325	0.548∼6.224	0.344				
MMR index (dMMR vs. pMMR)	1.169	0.163∼0.855	0.057				
Histological differentiation (Poor vs. Well)	3.266	0.061∼0.706	0.135				
Lymph node metastasis (N vs. P)	0.20	0.012∼0.219	0.001**		0.024	0.001∼0.686	0.029*
Distant metastasis (N vs. P)	1.625	2.537∼24.339	0.001**		6.560	3.299∼32.566	0.012*
HOTAIR expression (High vs. Low)	0.152	0.114∼0.562	0.012**		0.188	0.038∼0.757	0.017*

HR, hazard ratio; N, negative; P, positive; *P<0.05, **P<0.01.

## Abbreviations

CRC: Colorectal cancer
lncRNAs: long noncoding RNAs
HOTAIR: Homeobox transcript antisense inter-genic RNA
ceRNA: Competing endogenous RNA
miRNA: microRNA
qRT-PCR: Quantitative real-time polymerase chain reaction
OS: overall survival
PFS: progression-free survival

## References

[1] Miller KD, Siegel RL, Lin CC, Mariotto AB, Kramer JL, Rowland JH (2016). Cancer treatment and survivorship statistics, 2016. CA: a cancer journal for clinicians.

[2] Chen W, Zheng R, Baade PD, Zhang S, Zeng H, Bray F (2016). Cancer statistics in China, 2015. CA: a cancer journal for clinicians.

[3] DeSantis CE, Lin CC, Mariotto AB, Siegel RL, Stein KD, Kramer JL (2014). Cancer treatment and survivorship statistics, 2014. CA: a cancer journal for clinicians.

[4] Li X, Wu Z, Fu X, Han W (2013). Long Noncoding RNAs: Insights from Biological Features and Functions to Diseases. Medicinal research reviews.

[5] Kita Y, Yonemori K, Osako Y, Baba K, Mori S, Maemura K (2017). Noncoding RNA and colorectal cancer: its epigenetic role. Journal of human genetics.

[6] Gupta RA, Shah N, Wang KC, Kim J, Horlings HM, Wong DJ (2010). Long non-coding RNA HOTAIR reprograms chromatin state to promote cancer metastasis. Nature.

[7] Tatangelo F, Di Mauro A, Scognamiglio G, Aquino G, Lettiero A, Delrio P (2018). Posterior HOX genes and HOTAIR expression in the proximal and distal colon cancer pathogenesis. Journal of translational medicine.

[8] Xiao Z, Qu Z, Chen Z, Fang Z, Zhou K, Huang Z (2018). LncRNA HOTAIR is a Prognostic Biomarker for the Proliferation and Chemoresistance of Colorectal Cancer via MiR-203a-3p-Mediated Wnt/ß-Catenin Signaling Pathway. Cellular physiology and biochemistry: international journal of experimental cellular physiology, biochemistry, and pharmacology.

[9] Li P, Zhang X, Wang L, Du L, Yang Y, Liu T (2017). lncRNA HOTAIR Contributes to 5FU Resistance through Suppressing miR-218 and Activating NF-κB/TS Signaling in Colorectal Cancer. Molecular therapy. Nucleic acids.

[10] Pan S, Liu Y, Liu Q, Xiao Y, Liu B, Ren X (2019). HOTAIR/miR-326/FUT6 axis facilitates colorectal cancer progression through regulating fucosylation of CD44 via PI3K/AKT/mTOR pathway. Biochimica et biophysica acta. Molecular cell research.

[11] Lu X, Liu Z, Ning X, Huang L, Jiang B (2018). The Long Noncoding RNA HOTAIR Promotes Colorectal Cancer Progression by Sponging miR-197. Oncology research.

[12] Huang Y, Lee D, Young C (2020). Predictors for complete pathological response for stage II and III rectal cancer following neoadjuvant therapy - A systematic review and meta-analysis. American journal of surgery.

[13] Bregni G, Akin Telli T, Camera S, Deleporte A, Moretti L, Bali AM (2020). Adjuvant chemotherapy for rectal cancer: Current evidence and recommendations for clinical practice. Cancer treatment reviews.

[14] Simillis C, Singh H, Afxentiou T, Mills S, Warren OJ, Smith JJ (2020). Postoperative chemotherapy improves survival in patients with resected high-risk Stage II colorectal cancer: results of a systematic review and meta-analysis. Colorectal disease: the official journal of the Association of Coloproctology of Great Britain and Ireland.

[15] Ye J, Luo Y, Fang W, Pan J, Zhang Z, Zhang Y (2015). Real-time cell analysis for monitoring cholera toxin-induced human intestinal epithelial cell response. Current microbiology.

[16] Xu C, Zheng L, Li D, Chen G, Gu J, Chen J (2018). CXCR4 overexpression is correlated with poor prognosis in colorectal cancer. Life sciences.

[17] Botti G, Marra L, Malzone MG, Anniciello A, Botti C, Franco R (2017). LncRNA HOTAIR as Prognostic Circulating Marker and Potential Therapeutic Target in Patients with Tumor Diseases. Current drug targets.

[18] Pádua Alves C, Fonseca AS, Muys BR, de Barros E Lima Bueno R, Bürger MC, de Souza JE (2013). Brief report: The lincRNA Hotair is required for epithelial-to-mesenchymal transition and stemness maintenance of cancer cell lines. Stem cells.

[19] Rinn JL, Kertesz M, Wang JK, Squazzo SL, Xu X, Brugmann SA (2007). Functional demarcation of active and silent chromatin domains in human HOX loci by noncoding RNAs. Cell.

[20] Liu LC, Wang YL, Lin PL, Zhang X, Cheng WC, Liu SH (2019). Long non-coding RNA HOTAIR promotes invasion of breast cancer cells through chondroitin sulfotransferase CHST15. International journal of cancer.

[21] Kim K, Jutooru I, Chadalapaka G, Johnson G, Frank J, Burghardt R (2013). HOTAIR is a negative prognostic factor and exhibits pro-oncogenic activity in pancreatic cancer. Oncogene.

[22] Chen FJ, Sun M, Li SQ, Wu QQ, Ji L, Liu ZL (2013). Upregulation of the long non-coding RNA HOTAIR promotes esophageal squamous cell carcinoma metastasis and poor prognosis. Molecular carcinogenesis.

[23] Gao JZ, Li J, DU JL, Li XL (2016). Long non-coding RNA HOTAIR is a marker for hepatocellular carcinoma progression and tumor recurrence. Oncology letters.

[24] Huang KB, Zhang SP, Zhu YJ, Guo CH, Yang M, Liu J (2019). Hotair mediates tumorigenesis through recruiting EZH2 in colorectal cancer. Journal of cellular biochemistry.

[25] Svoboda M, Slyskova J, Schneiderova M, Makovicky P, Bielik L, Levy M (2014). HOTAIR long non-coding RNA is a negative prognostic factor not only in primary tumors, but also in the blood of colorectal cancer patients. Carcinogenesis.

[26] Huang X, Lu S (2017). MicroR-545 mediates colorectal cancer cells proliferation through up-regulating epidermal growth factor receptor expression in HOTAIR long non-coding RNA dependent. Molecular and cellular biochemistry.

[27] Samaeekia R, Adorno-Cruz V, Bockhorn J, Chang YF, Huang S, Prat A (2017). miR-206 Inhibits Stemness and Metastasis of Breast Cancer by Targeting MKL1/IL11 Pathway. Clinical cancer research: an official journal of the American Association for Cancer Research.

[28] Xiao H, Xiao W, Cao J, Li H, Guan W, Guo X (2016). miR-206 functions as a novel cell cycle regulator and tumor suppressor in clear-cell renal cell carcinoma. Cancer letters.

[29] Yang Y, Wang W, Chang H, Han Z, Yu X, Zhang T (2019). Reciprocal regulation of miR-206 and IL-6/STAT3 pathway mediates IL6-induced gefitinib resistance in EGFR-mutant lung cancer cells. Journal of cellular and molecular medicine.

[30] Meng X, Fu R (2018). miR-206 regulates 5-FU resistance by targeting Bcl-2 in colon cancer cells. OncoTargets and therapy.

[31] Deng M, Qin Y, Chen X, Wang Q, Wang J (2019). MiR-206 inhibits proliferation, migration, and invasion of gastric cancer cells by targeting the MUC1 gene. OncoTargets and therapy.

[32] Ren XL, He GY, Li XM, Men H, Yi LZ, Lu GF (2016). MicroRNA-206 functions as a tumor suppressor in colorectal cancer by targeting FMNL2. Journal of cancer research and clinical oncology.

[33] Li T, Qin Y, Zhen Z, Shen H, Cong T, Schiferle E (2019). Long non-coding RNA HOTAIR/microRNA-206 sponge regulates STC2 and further influences cell biological functions in head and neck squamous cell carcinoma. Cell proliferation.

[34] Chang L, Guo R, Yuan Z, Shi H, Zhang D (2018). LncRNA HOTAIR Regulates CCND1 and CCND2 Expression by Sponging miR-206 in Ovarian Cancer. Cellular physiology and biochemistry: international journal of experimental cellular physiology, biochemistry, and pharmacology.

[35] Ding W, Ren J, Ren H, Wang D (2017). Long Noncoding RNA HOTAIR Modulates MiR-206-mediated Bcl-w Signaling to Facilitate Cell Proliferation in Breast Cancer. Scientific reports.

[36] Shen H, Yu X, Yang F, Zhang Z, Shen J, Sun J (2016). Reprogramming of Normal Fibroblasts into Cancer-Associated Fibroblasts by miRNAs-Mediated CCL2/VEGFA Signaling. PLoS genetics.

[37] Lewis C, Murdoch C (2005). Macrophage responses to hypoxia: implications for tumor progression and anti-cancer therapies. The American journal of pathology.

[38] Kuroda T, Kitadai Y, Tanaka S, Yang X, Mukaida N, Yoshihara M (2005). Monocyte chemoattractant protein-1 transfection induces angiogenesis and tumorigenesis of gastric carcinoma in nude mice via macrophage recruitment. Clinical cancer research: an official journal of the American Association for Cancer Research.

[39] Liu J, Chen S, Wang W, Ning BF, Chen F, Shen W (2016). Cancer-associated fibroblasts promote hepatocellular carcinoma metastasis through chemokine-activated hedgehog and TGF-β pathways. Cancer letters.

