# Supplementary material for: Long non-coding RNA HOTAIR as a competitive endogenous RNA to sponge miR-206 to promote colorectal cancer progression by activating CCL2
Source: J Cancer. 2020 May 18;11(15):4431–41. doi: 10.7150/jca.42308 (PMC7255378; doi:10.7150/jca.42308)
Supplement: Supplementary file 1 — Supplementary tables. [file jcav11p4431s1.pdf]

**Table S1. Complete list of primers used in this work. Sequences are from the GenBank.**

| Name | sequencing |
|---|---|
| CCL2 | Forward: GAGGGCGACACTGCTTTTTC |
| | Reverse: CCAGCTCCAGGAAATGCTAG |
| HOTAIR | Forward: GGTAGAAAAAGCAACCACGAAGC |
| | Reverse: ACATAAACCT CTGTCTGTG AGTGCC |
| GAPDH | Forward: CACCCACTCCTCCACCTTTG |
| | Reverse: CCACCACCCTGTTGCTGTAG |
| miR-206 | Forward:5'-CCACACACTTCCTTACATTCCA-3' |
| | Reverse:5'-GCGAGCACAGAATTAATACGAC-3' |
| U6 | Forward: 5'-CTCGCTTCGGCAGCACA-3' |
| | Reverse: 5'-AACGCTTCACGAATTTGCGT-3' |
| Si-HOTAIR | #1:5'-GAACGGGAGUACAGAGAGAUU-3' |
| | #2:5'-CCACAUGAACGCCCAGAGAUU-3' |
| Si-NC | 5'-CUACAACAGCCACAACGUCdTd-3' |

**Table S2. Primary antibodies for Immunohistochemistry and Western-blot and IHC**

| primary antibody | Details |
|---|---|
| CCL2 | mouse polyclonal,BF0556,(Affinity, Cincinnati, OH, USA) |
| GAPDH | Rabbit monoclonal,ab181602,(abcam,Cambridge, MA, USA) |
